# Population Genetics Reveals the Invasion Pathways of *Mesembryanthemum nodiflorum* in North America

**DOI:** 10.1002/ece3.72262

**Published:** 2025-10-15

**Authors:** Clarke J. M. van Steenderen, Emma Sandenbergh, Dean Brookes, Patrick J. Moran, Massimo Cristofaro, William F. Hoyer, Iain D. Paterson

**Affiliations:** ^1^ Department of Zoology and Entomology, Centre for Biological Control Rhodes University Makhanda Eastern Cape South Africa; ^2^ Commonwealth Scientific and Industrial Research Organisation (CSIRO), Queensland Brisbane Queensland Australia; ^3^ U.S. Department of Agriculture, Agricultural Research Service (USDA‐ARS), Invasive Species and Pollinator Research Unit (ISPHRU), California Albany California USA; ^4^ Biotechnology and Biological Control Agency (BBCA) Rome Italy; ^5^ Naval Base Ventura County Point Mugu California USA

**Keywords:** biological control, bridgehead, ice plant, invasion routes, invasive species, ISSR, RADseq

## Abstract

Invasive populations of the slenderleaf iceplant, *Mesembryanthemum nodiflorum* L., are problematic along the west coast of North America. The plant is hypothesised to originate from southern Africa, though it has established populations in North Africa and the Mediterranean. There is interest in initiating a biological control programme for the weed in its invaded range, but a clearer understanding of its invasion pathways and sources of origin is required in order to prioritise potential biological control agents. This study used both NextGen RADseq and fragment analysis ISSR techniques to uncover the population structure and genetic diversity of *M. nodiflorum* in its native, introduced and invaded ranges. The results supported a South African origin of the species based on a higher number of private alleles and overall genetic diversity. Our results suggest a bridgehead effect, where a secondary invasion to North America from Mediterranean populations took place, rather than a direct invasion from the native range in South Africa. The present results indicated that surveys for potential biocontrol agents for *M. nodiflorum* should be conducted in the native South African distribution, where the greatest diversity of specialist natural enemies is likely to be present.

## Graphical abstract

*Mesembryanthemum nodiflorum* is a species of ice plant native to South Africa that has become invasive in California and Mexico. The present study used RADseq data to run a population genetics analysis to match the invasive populations to those in the native and introduced ranges of the Mediterranean. The results supported a secondary invasion of the plant from the introduced populations in the Mediterranean to North America, and that surveys for biological control agents should focus on the native range in South Africa.

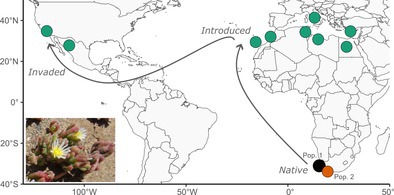

## 1 Introduction

Invasive plants are a leading cause of biodiversity loss globally, causing major ecological and economic damage (Mack et al. 2000; Ehrenfeld 2010; Simberloff et al. 2013; Blackburn et al. 2014; Pyšek et al. 2020; Diagne et al. 2021; Macêdo et al. 2024). One such invasive species, *Mesembryanthemum nodiflorum* L. (Aizoaceae: Mesembryanthemoideae) (the slenderleaf iceplant), exemplifies these challenges due to its rapid spread and ecological impacts (Olckers et al. 2021; De La Cruz et al. 2023). Control methods for invasive plants typically include repeated herbicide applications, mechanical removal and burning, but the costs involved can become substantial (Van Wyk and Van Wilgen 2002; Culliney 2005; Pimentel et al. 2005; Maluleke et al. 2021). Additionally, the negative nontarget impacts of herbicides on the environment (Relyea 2005; Wagner and Nelson 2014; Peterson et al. 2018), and the development of herbicidal resistance (Busi et al. 2013; Damalas and Koutroubas 2024), are well‐documented.

Biological control (hereafter ‘biocontrol’) is a safe, cost‐effective management strategy for the control of invasive taxa through the use of their natural enemies (‘biocontrol agents’) (McFadyen 1998; Stenberg et al. 2021; Schwarzländer et al. 2018). The safety and success of a biocontrol programme are dependent on the selection of suitably damaging natural enemies that are host‐specific and that will survive and reproduce successfully in their host's invaded range (Van Klinken and Edwards 2002; Stiling and Cornelissen 2005; McEvoy 2018).

A number of studies in the biocontrol literature have focused on matching invasive plant populations to their source of origin in order to streamline the selection of candidate biocontrol agents that have coevolved with the target weed (Goolsby et al. 2006; Paterson et al. 2009; Sutton et al. 2017; Tanga et al. 2021; Mitchell et al. 2024). The present study used population genetic tools to identify the source(s) of origin of the invasive North American populations of *M. nodiflorum* . Although this annual plant species has a cosmopolitan distribution, there is evidence to support a southern African origin for the genus (Jacobsen and Burman 1956; Kloot 1983; Klak et al. 2003; Gutterman and Gendler 2005; Gerbaulet and Hartmann 2017).

Based on a phylogenetic analysis of the family, Klak et al. (2003) concluded that the majority of the Aizoaceae originate from the arid regions of southern Africa and that nearly all known taxa in the Mesembryanthemoideae are endemic to the region. *Mesembryanthemum nodiflorum* is considered indigenous in the Northern Cape and Western Cape provinces of South Africa (Gerbaulet and Hartmann 2017), while its status in the Mediterranean is disputed (Royal Botanic Gardens, Kew 2025; Pladias Database Team 2025). The plant is also found from Iran through the Arabian Peninsula, southern Europe and northern Africa and is invasive in Mexico, California and south‐western Australia (Chinnock et al. 2012; Gerbaulet and Hartmann 2017). *Mesembryanthemum nodiflorum* appears in a list of alien flora in Turkey, where it is classified as an ‘archaeophyte’ (an alien species introduced to an area in ancient times by humans) (Uludağ et al. 2017). The plant has become particularly problematic along the west coast of North America, with large infestations in California (USA) and Mexico (Royal Botanic Gardens, Kew 2025). The plant invades arid, high‐salinity areas, where it tends to exclude native species through the release of salts into the surrounding soil after the death of the plant (De La Cruz et al. 2023).

A growing number of population genetics studies in invasion biology are applying restriction site‐associated DNA sequencing (RADseq) techniques in order to uncover finer‐scale insights into the genetic structuring and cryptic variation of invasive species (Jeffery et al. 2017; Baltazar‐Soares et al. 2020; McCartney et al. 2019; Li et al. 2024). RADseq methods have become increasingly popular due to their relatively low cost, large data output for single nucleotide polymorphism (SNP) discovery and genotyping, and their applicability to nonmodel organisms (Davey and Blaxter 2010; Peterson et al. 2012; Andrews et al. 2016). The use of intersimple sequence repeats (ISSRs) is an older genotyping method that relies on the comparison of the fragment sizes of repeated nucleotide motifs located between microsatellite regions throughout the genome (Abbot 2001; Wolfe 2005). The method was initially developed to differentiate between crop cultivars, but, since the primers are universal, it has been applied across a range of taxa (Wolfe 2005).

**FIGURE 1:**
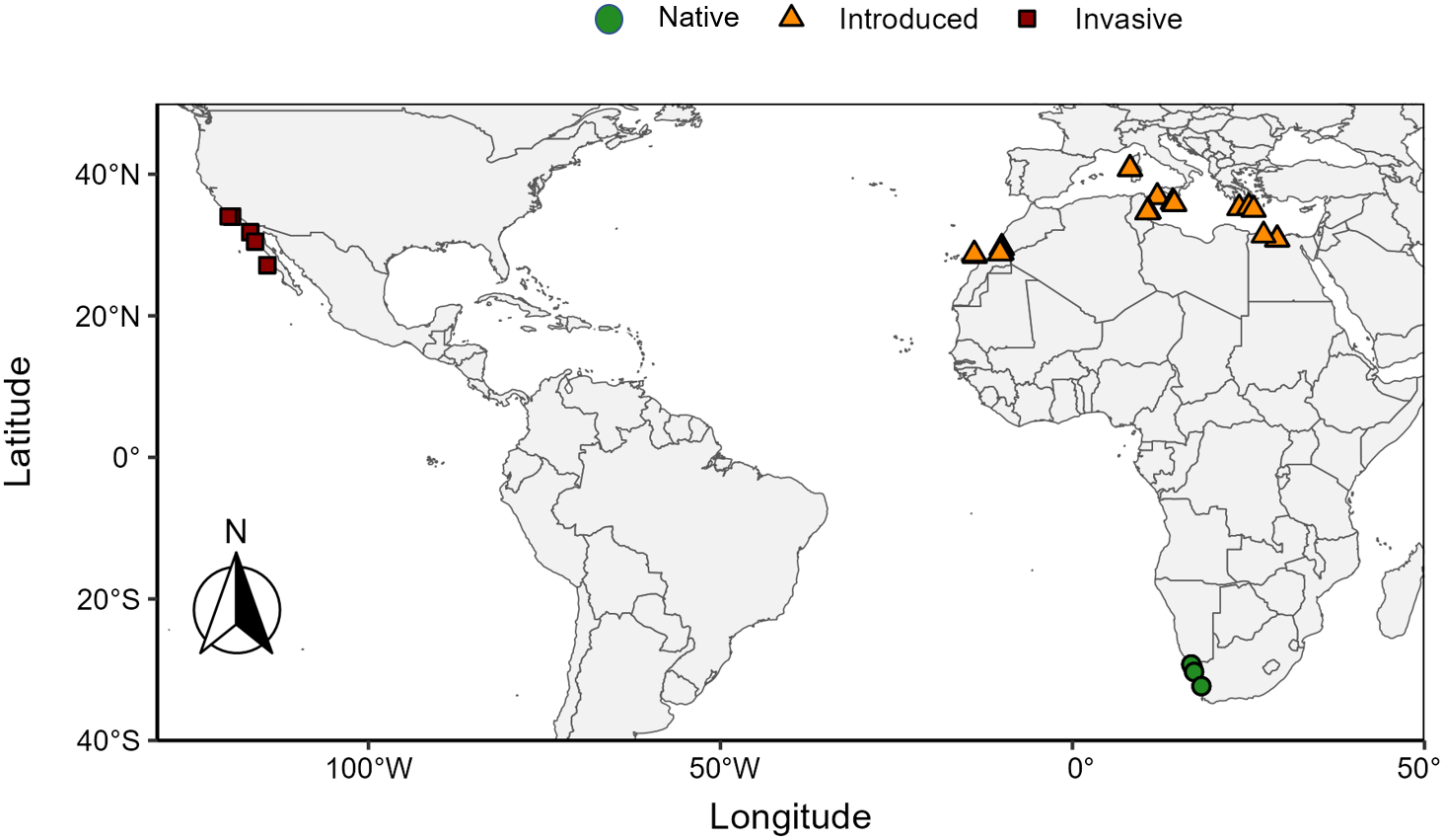
*Mesembryanthemum nodiflorum* sample collection for the present study, showing the plant's native range in South Africa (green circles), introduced range in North Africa and the Mediterranean (orange triangles), and the invaded range in California and Mexico (dark red squares).

Here, both a RADseq and an ISSR fragment analysis method were applied to *M. nodiflorum* samples collected across its native, introduced and invaded range (Figure 1). The aims were to (1) target geographic areas for field surveys and the collection of candidate biocontrol agents and (2) compare the outputs of a modern (RADseq) and traditional (ISSR) analysis of the same data.

To date, current surveys in the native range have found at least two beetle species (Coleoptera: Urodontidae) that feed on the fruits, stems and crowns of *M. nodiflorum* . These urodontids have been identified as *Urodontus mesemoides* Louw and *U. tessallatus* Louw, and show promise as potential biological control agents (unpublished data). The results of the present work will have important applications to the initiation of a biological control programme for *M. nodiflorum* in its invaded range and will add to the limited available literature on the biogeography and genetic structure of this halophyte.

## 2 Materials and Methods

### 2.1 Sample Collection and Laboratory Protocols

A total of 84 *Mesembryanthemum nodiflorum* samples were sourced from selected areas of the plant's native (*n* = 15; four sites), introduced (*n* = 45; 17 sites) and invaded range (*n* = 24; five sites) (Figure 1; File S1). The sites represent all the regions where the plant is present, with the exception of Iran, the Arabian Peninsula and Australia. The four sites within South Africa were spread across the majority of the plant's distribution in the country.

#### 2.1.1 DNA Extraction

DNA extractions were performed using an Invitrogen PureLink Genomic Plant DNA Purification Kit according to the manufacturer's protocols. Plant material was crushed under liquid nitrogen, and final extracts were stored at −40°C. All starting plant DNA extracts were quantified using a ThermoFisher Scientific Qubit dsDNA High Sensitivity Assay Kit and Qubit 4 Fluorometer and normalised to concentration pools of < 1, 1, 2, 3, 4 and 6 ng/μL.

#### 2.1.2 RADseq Protocols

The Adapterama III methodology (Bayona‐Vásquez et al. 2019) was used to prepare RADseq libraries. Sequencing and size selection were performed by Admera Health (https://www.admerahealth.com/) in the United States of America.

##### 2.1.2.1 Internal Index Preparation

The ClaI and EcoRI iTru i5 and iTru i7 internal indexes, respectively, were utilised in this study (Bayona‐Vásquez et al. 2019). All internal index primers were combined according to their corresponding upper and lower stubs in equal volumes such that the final stock concentration was 50 μM. Corresponding upper and lower stubs were annealed through a PCR protocol set to 95°C for 1 min, followed by cooling by 0.1°C per second until room temperature was reached.

##### 2.1.2.2 Restriction Digestion and Ligation

Restriction digestion was performed using the MspI read 1 and EcoRI‐HF read 2 enzymes, and the ClaI read 1 adapter dimer‐cutting enzyme as per Table A1. Constituents were incubated in a thermal cycler for 1 h at 37°C. Following restriction digestion, a ligation step was followed according to Table A2. A PCR protocol was subsequently run that involved incubation at 22°C for 20 min; 37°C for 10 min; 22°C for 20 min; 37°C for 10 min; 80°C for 20 min; and hold at 10°C.

##### 2.1.2.3 Pooling and Clean‐Up

Following ligation, a 10 μL volume of each sample was used for pooling according to the predefined concentration categories. Pooled sample volumes were cleaned and size‐selected using AmPure XP beads at a ratio of 1:1.25 according to the manufacturer's protocols. dH_2_O was used for the final elution.

##### 2.1.2.4 Library PCR

The external index pair iTru5_01_A and iTru7_101_01 were added in the final library PCR preparation step as per Table A3. The PCR protocol was repeated in triplicate and entailed 98°C for 30 s; 20 cycles of 98°C for 10 s, 60°C for 15 s, 72°C for 30 s; 72°C for 5 min; and hold at 10°C. Triplicate PCR products were subsequently recombined back into one solution. Libraries were cleaned in a final bead clean‐up step, as detailed above. Products were checked on a 1% agarose gel before proceeding with sequencing.

##### 2.1.2.5 Sequencing

Fragments were size‐selected to 525 bp ±10% using a Blue Pippin, following sequencing on a NovaSeq X plus 10B 2 × 150 instrument. Samples were processed on 1 lane, with 2–2.5B PE per lane (1–1.250B in each direction). Data were shared using BaseSpace, and all subsequent bioinformatic analyses were performed on a Linux system via the Centre for High Performance Computing (CHPC) platform hosted by the South African Council for Scientific and Industrial Research (CSIR).

### 2.2 RADseq Bioinformatics

All samples were checked for quality using FastQC (Andrews 2010). Sample files were demultiplexed using the *process_radtags* function in Stacks (Catchen et al. 2013), where final read lengths were truncated to 140 bp. Failed reads were discarded using the *filter‐illumina* parameter, and *barcode‐dist* was set to 2. R scripts were written to assist with a standardised approach for generating input files for internal index and population assignment information.

As there was no available reference genome for *M. nodiflorum* , the Stacks *denovo_map.pl* function was employed. In the case of large data files, rather than running the *denovo_map.pl* wrapper function, samples were run in batches of 10 at a time using *ustacks*. The full output was then passed through the *cstacks* →*sstacks* → *tsv2bam* → *gstacks* → *populations* pipeline as individual modules. Further SNP filtering was carried out using the *SNPfiltR* v 1.0.2 (DeRaad 2022) and *vcfR* v 1.15.0 (Knaus and Grünwald 2017) packages in R (R Core Team 2025; Posit Team 2025). The *dartR* v 2.9.7 package (Gruber et al. 2018) was used to convert vcf files into fastSTRUCTURE format.

SNPs were filtered downstream such that data with a genotype quality value less than 35 (3.28% of the data) and a depth less than 5 (4.34% of the data) were discarded. A further 11.9% of heterozygous genotypes were discarded following an allele balance ratio check. Next, SNPs with a mean depth above 125 were removed, equating to 19.92% of the data. Missing data were then assessed for each sample, where a cut‐off value of 0.9 was applied. This cut‐off removed 13 samples from the data set. Thereafter, SNPs that fell below a minor allele count (MAC) of 1 were removed (17.33% of the data). A final filtering step was applied where missing data per SNP were removed, using a cut‐off value of 0.8 (91.48% SNPs removed).

The fastSTRUCTURE software was preferred as this approach is able to process much larger data sets, while the traditional STRUCTURE software (Pritchard et al. 2000) poses computational limitations (Wang 2022). Here, *K*‐values between 1 and 10 were tested for fastSTRUCTURE. The software does not take any a priori groupings and is therefore an unsupervised clustering method.

A principal component analysis (PCA) was run using the *adegenet* v 2.1.11 (Jombart 2008) R package. Population statistics were generated using the *poppr* v 2.9.6 and *hierfstat* v 0.5‐11 (*F*_ST_ values) packages, where population assignments were made on (1) its status as native, introduced or invasive (‘broad’), (2) the country of origin for each sample (‘country’) and (3) specific collection sites (‘site’). Confidence intervals for *F*_ST_ values were calculated using the *boot.ppfst()* function in the *hierfstat* package, applying 10,000 bootstrap repeats. The fixation index (*F*_IS_) measures the degree of inbreeding in a population, and therefore indicates the level of gene flow present (Nei and Chesser 1983). The index of association (*I*_*A*_ and the standardised $\bar{r}d$) is a measure of asexual or clonal reproduction in a population, quantified by the frequency of linked loci present (Brown et al. 1980). The *G* (Stoddard and Taylor's Index), *H* (Shannon–Weiner Index) and lambda (Simpson's Index) diversity indices are different measures of SNP diversity within populations. Private alleles (*PA*) refer to unique alleles found only in a particular population. All Linux job scripts and associated R coding files are stored on a GitHub repository (https://github.com/clarkevansteenderen/RADseq_pipeline) and permanent Zenodo link (doi: 10.5281/zenodo.17044994), where tailored Stacks parameters are provided. Data are available on the NCBI Short Read Archive (SRA) under project ID PRJNA1284890.

#### 2.2.1 Intersimple Sequence Repeat (ISSR) Analysis

All 84 samples were processed for ISSR analysis, using universal ISSR primers HB13 (${\left[\mathrm{GAG}\right]}_{3}\mathrm{GC}$) and HB15 (${\left[\mathrm{GTG}\right]}_{3}\mathrm{GC}$) (Wolfe et al. 1998) that were labelled with 5′6‐FAM fluorescent dye. These two primers optimised the number of bands produced and have been used in other plant studies (Paterson et al. 2009; Soliman et al. 2014; Reid et al. 2021). PCRs for ISSR samples were run in 20 μL reactions, consisting of 10 μL Universal SYBR Green Supermix (Bio‐Rad), 0.8 μM primer, 6.2 μL denucleated water and 3 μL DNA template. All PCR reactions were replicated as per Paterson et al. (2009); van Steenderen et al. (2021); Reid et al. (2021). The PCR protocol was followed as per Table A4.

Samples were analysed at the Central Analytical Facilities (CAF) division in Stellenbosch, South Africa, where DNA fragment analysis was performed using capillary electrophoresis (Applied Biosystems Inc., 3130 genetic analyser, GS1200LIZ size standard).

Electropherograms were analysed in Genemarker v 2.7.4 (SoftGenetics). Binary data from GeneMarker were opened in RawGeno v 2.0 (Arrigo et al. 2012), where maximum and minimum bin widths were set to 1 and 0.5 base pairs (bp), respectively; scoring ranged from 100 to 500 bp, and a low relative fluorescent units (RFU) of 100 was applied. After filtering for quality, 51 samples were retained for further analysis, comprising samples from the indigenous (*n* = 9; three sites), introduced (*n* = 26; 11 sites) and invaded ranges (*n* = 16; five sites).

Binary output for each replicate sample pair for each individual primer was consolidated using BinMat software (van Steenderen 2022). Consolidated matrices for HB13 and HB15 were subsequently joined (total loci = 634) and used to create a NeighborNet tree in SplitsTree v 4, applying Jaccard's distance. STRUCTURE (Pritchard et al. 2000) was employed to further assess population structure, applying the admixture and recessive allele models, with uncorrelated allele frequencies (Stift et al. 2019). *K*‐values were tested between 2 and 5, and ploidy was set to 1. Burnin was set to 100,000 with MCMC repeats of 50,000. The remaining settings were left at their defaults. StructureSelector (Kopelman et al. 2015) was used to assess the optimal *K*‐value under both the broad and country‐grouping schemes.

The Jaccard Similarity Index was used to assess diversity within and between populations. An analysis of molecular variance (AMOVA) was run with hierarchical levels using the *adegenet* (Jombart 2008) and *poppr* R packages (Kamvar et al. 2014) in order to determine whether genetic variation could be explained by the assigned groupings. The AMOVA took the form: poppr.amova (issr.data, Broad/Country).

## 3 Results

### 3.1 RADseq Output

All 84 *Mesembryanthemum nodiflorum* samples were sequenced successfully, yielding a total of 2,557,839,842 fragment sequences. Of these, 65.4% of reads were retained after demultiplexing (1,671,699,816). The demultiplexing step reported 6.6% dropped reads due to unidentified barcodes, and 0.6% due to low quality. The analysis reported 27.4% missing RAD cut sites. Following assembly, 1,393,761 loci were genotyped, with a mean per‐sample depth coverage of 87× ± 48.4× (min = 4.7×, max = 214.9×). The mean number of sites per locus was 184.3.

Following initial filtering in Stacks' *populations* module, 83 samples remained with a total of 86,274 SNPs and 69% missing data. After more rigorous downstream filtering in *SNPfiltR*, the final data set contained 70 samples with a total of 4862 SNPs and 8.6% missing data (File S1).

### 3.2 Population Structure

**FIGURE 2:**
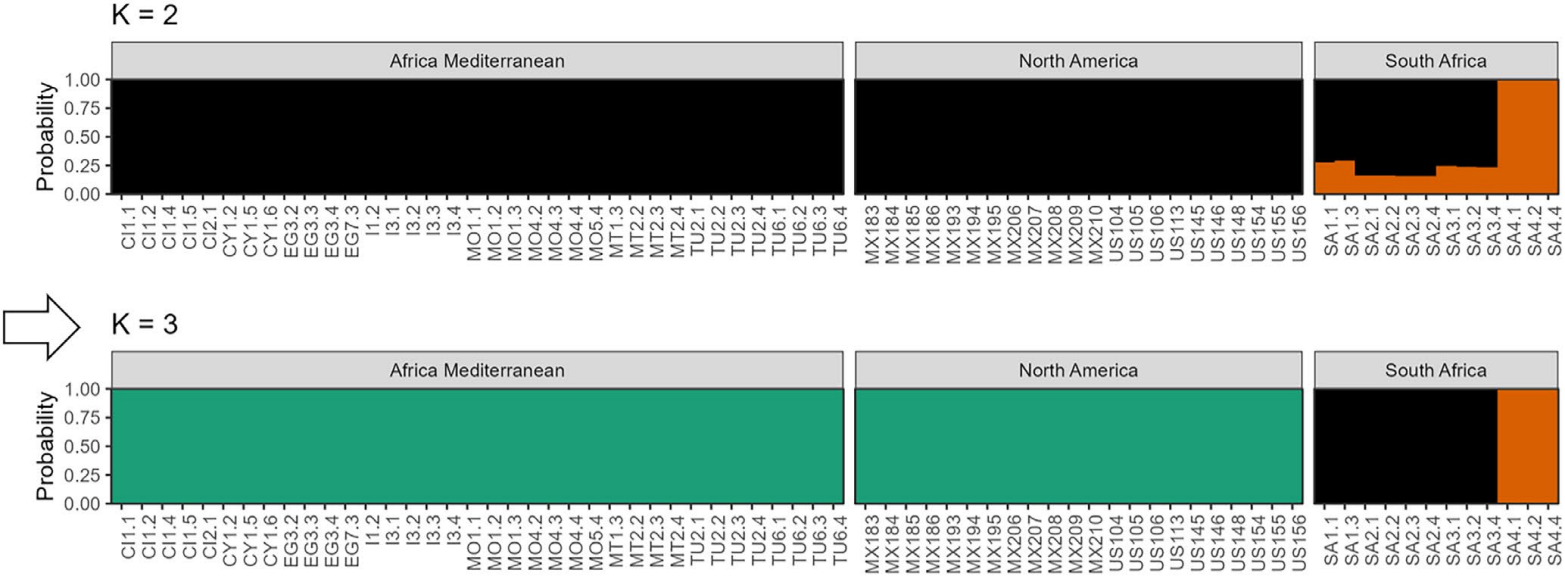
fastSTRUCTURE plots produced from the RADseq SNP data, where groups were: Introduced, North Africa and the Mediterranean; Invaded, California and Mexico; Native, South Africa. Outputs are shown for *K* = 2 and *K* = 3, where *K* = 3 was the optimal value that best explained population structure that best explained population structure (indicated by an arrow).

**FIGURE 3:**
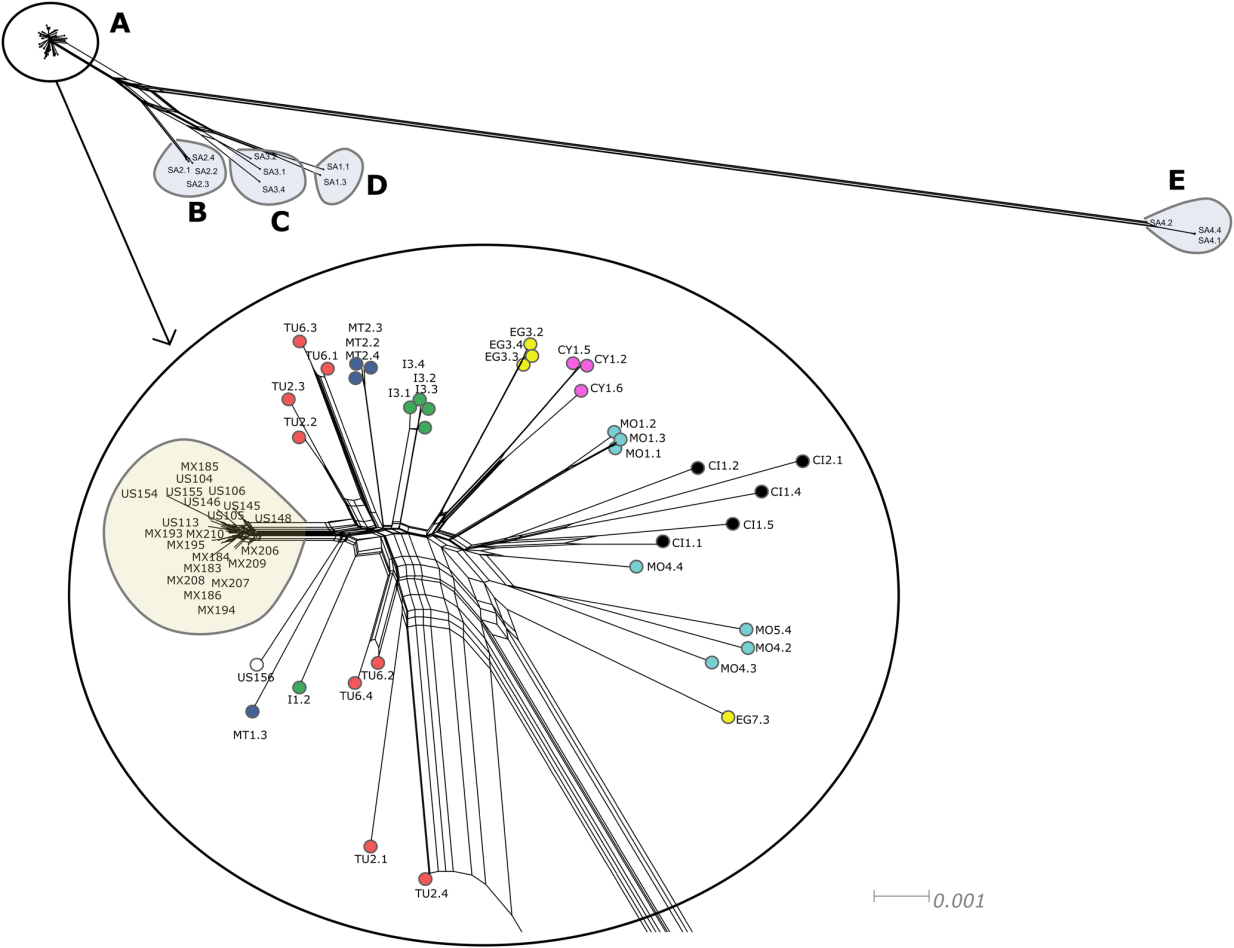
SplitsTree NeighbourNet diagram produced from the RADseq SNP data. Clade A contains all the invaded and introduced range samples and is enlarged for clarity. Clades B, C, D and E are populations in the native South African range. Sample IDs are detailed in File S1, where: CI, Canary Islands; CY, Cyprus; EG, Egypt; I, Italy; MO, Morocco; MT, Malta; MX, Mexico; SA, South Africa; TU, Tunisia; US, United States of America.

**FIGURE 4:**
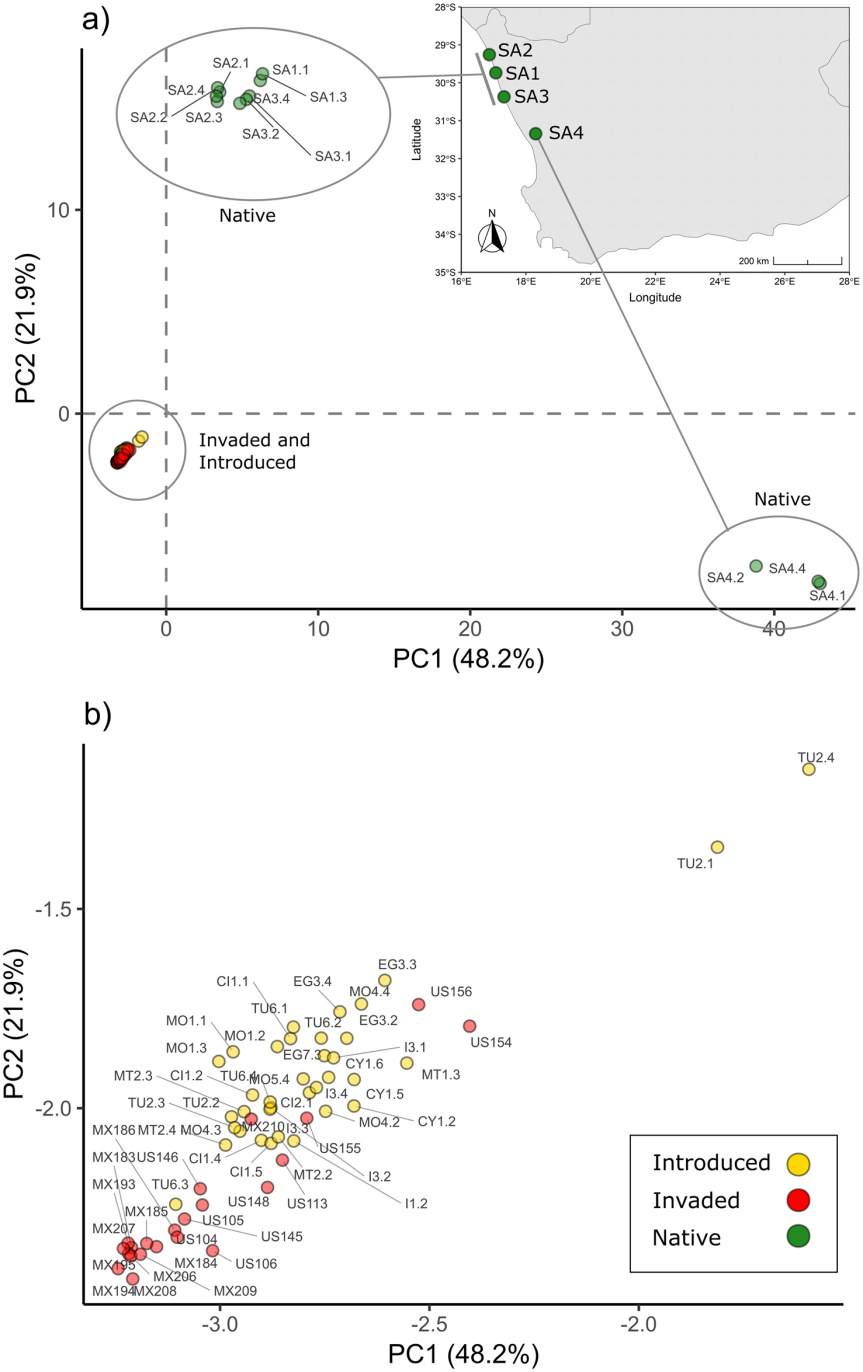
PCA results from the RADseq SNP data, showing (a) all samples with an inset map of collection sites in the native South African range, and (b) the same analysis, but showing only introduced and invaded range samples. CI, Canary Islands; CY, Cyprus; EG, Egypt; I, Italy; MT, Malta; MO, Morocco; MX, Mexico; SA, South Africa; TU, Tunisia; US, United States of America (California).

The fastSTRUCTURE results indicated that *K* = 3 best explained the genetic structure in the data set and maximised marginal likelihood. Under the *K* = 3 grouping structure, the North American invaded range matched the North African and Mediterranean samples, while the native South African samples were genotypically distinct (Figures 2, 3, 4; Table 1). The native range samples from South Africa (SA) formed two distinct genetic clusters; one comprising sites SA1, SA2 and SA3, and the second comprising site SA4 (Figures 2, 3, 4). Under the *K* = 2 grouping structure, native range sites SA1, SA2 and SA3 were most similar to those of the introduced and invaded ranges (Figure 2).

**TABLE 1:** *F*_ST_ pairwise comparisons across samples grouped by broad distribution.

	Introduced	Invaded	Native
Introduced		0.045/0.079	0.40/0.43
Invaded	0.062		0.39/0.42
Native	0.42	0.41	

*Note:* Values below the diagonal are *F*_ST_ values, and values above the diagonal are the lower and upper 95% confidence intervals, respectively, separated by a forward slash (/). Introduced, North Africa and the Mediterranean; Invaded, California and Mexico; Native, South Africa.

For the country‐level grouping approach, the *F*_ST_ values showed the highest similarity of the USA to Tunisia, Italy, Morocco and Malta (Table 2). The Mexican samples were most similar to those from Tunisia (Table 2), while the South African samples appeared as a unique genotype and were the most different from the Californian and Mexican samples (Table 2). The PCA showed a mild gradient between the Californian and Mexican samples and those from the Mediterranean, although the Mediterranean populations did not display significant structuring in that analysis (Figure 4b). The SplitsTree NeighbourNet analysis, however, showed the invaded populations clustering more closely to those from Tunisia, Malta and Italy (Figure 3).

**TABLE 2:** *F*_ST_ pairwise comparisons across samples grouped by country.

	CI	CY	EG	I	MO	MT	MX	SA	TU	US
CI		0.06/0.09	0.06/0.09	0.05/0.09	0.03/0.05	0.05/0.09	0.11/0.16	0.22/0.25	0.05/0.08	0.09/0.14
CY	0.07		0.05/0.09	0.06/0.11	0.04/0.07	0.06/0.1	0.11/0.16	0.15/0.18	0.05/0.09	0.09/0.14
EG	0.07	0.07		0.05/0.09	0.04/0.07	0.07/0.1	0.1/0.15	0.18/0.21	0.05/0.08	0.08/0.13
I	0.07	0.09	0.07		0.04/0.08	0.04/0.07	0.07/0.12	0.22/0.25	0.03/0.06	0.06/0.1
MO	0.04	0.06	0.06	0.06		0.04/0.07	0.09/0.14	0.25/0.28	0.04/0.07	0.07/0.12
MT	0.07	0.08	0.08	0.06	0.05		0.07/0.12	0.19/0.22	0.04/0.06	0.06/0.1
MX	0.13	0.13	0.13	0.09	0.11	0.10		0.34/0.36	0.05/0.09	0/0.01
SA	0.23	0.17	0.20	0.23	0.27	0.20	0.35		0.26/0.28	0.3/0.32
TU	0.07	0.07	0.07	0.04	0.05	0.05	0.07	0.27		0.04/0.08
US	0.11	0.12	0.11	0.08	0.09	0.08	0.00	0.31	0.06	

*Note:* Values below the diagonal are *F*_ST_ values, and values above the diagonal are the lower and upper 95% confidence intervals, respectively, separated by a forward slash (/).

Abbreviations: CI, Canary Islands; CY, Cyprus; EG, Egypt; I, Italy; MT, Malta; MO, Morocco; MX, Mexico; SA, South Africa; TU, Tunisia; US, United States of America (California).

**FIGURE 5:**
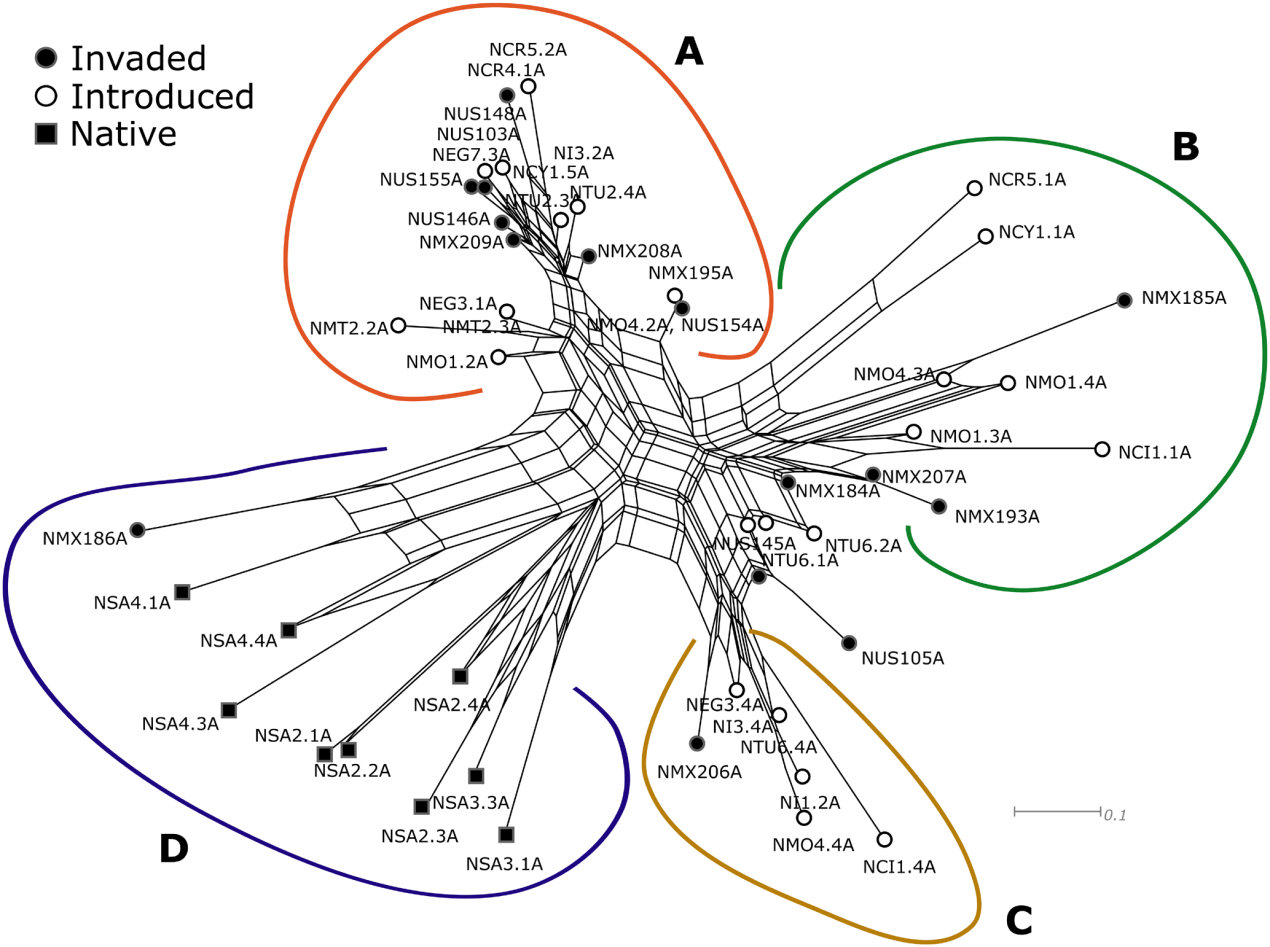
SplitsTree NeighbourNet diagram produced from the ISSR data, applying the Jaccard distance index, representing both the HB13 and HB15 primers. Clades A—C comprise samples from the invaded and introduced ranges, and clade D contains native South African samples with one Mexican sample. Sample IDs are detailed in File S1, where: NCI, Canary Islands; NCR, Crete; NCY, Cyprus; NEG, Egypt; NI, Italy; NMO, Morocco; NMT, Malta; NMX, Mexico; NSA, South Africa; NTU, Tunisia; NUS, United States of America.

**FIGURE 6:**
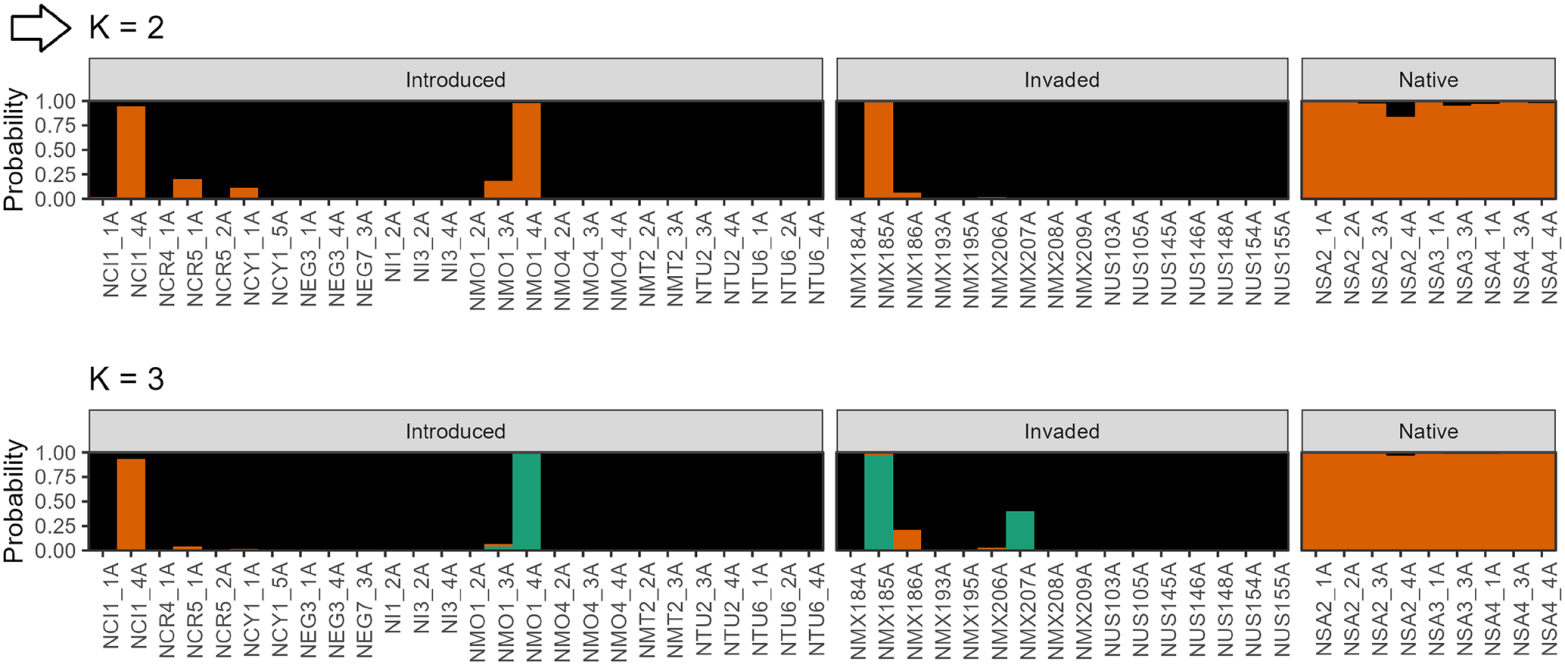
STRUCTURE output based on the ISSR data (HB13 + HB15 primers) for a broader grouping strategy, with *K* = 2 as the optimal value (indicated by an arrow). Output for *K* = 3 is shown additionally. Introduced, North Africa and the Mediterranean; Invaded, California and Mexico; Native, South Africa.

### 3.3 Population Statistics

Under the broad grouping scheme (invaded, introduced, and native range), the native range of South Africa showed the highest positive fixation index, index of association, allele richness and number of private alleles, and the lowest diversity indices and number of multilocus genotypes (Table 3). The observed heterozygosity for the native range was much lower than the expected heterozygosity, supporting the high inbreeding coefficient (*F*_IS_) reported. Conversely, the invaded range of California and Mexico showed the lowest fixation index, allele richness and number of private alleles (Table 3). The introduced range showed the highest diversity indices (Table 3). Both the introduced and invaded ranges showed higher observed heterozygosity values than expected heterozygosity, supporting the high degree of outbreeding reported.

**TABLE 3:** Population statistics under the three defined grouping schemes.

Group	*N*	*MLG*	*eMLG*	SE	*H*	*G*	Lambda	*I*_*A*_	$\bar{r}d$	$F_{\text{IS}}$	$H_{o}$	$H_{s}$	Allele richness	Private alleles
Broad														
Introduced	36.00	36.00	12.00	0.00	3.58	36.00	0.97	7.04	0.01	−0.13	0.13	0.08	1.18	664.00
Invaded	22.00	22.00	12.00	0.00	3.09	22.00	0.95	16.44	0.11	−0.77	0.12	0.06	1.13	61.00
Native	12.00	12.00	12.00	0.00	2.48	12.00	0.92	1147.21	0.34	0.81	0.03	0.29	1.74	3453.00
Country														
CI	5.00	5.00	5.00	0.00	1.61	5.00	0.80	3.67	0.02	−0.60	0.13	0.08	1.09	160.00
CY	3.00	3.00	3.00	0.00	1.10	3.00	0.67	26.93	0.49	−0.86	0.13	0.07	1.09	38.00
EG	4.00	4.00	4.00	0.00	1.39	4.00	0.75	81.53	0.74	−0.75	0.13	0.08	1.09	63.00
I	5.00	5.00	5.00	0.00	1.61	5.00	0.80	40.52	0.45	−0.82	0.13	0.07	1.08	28.00
MO	7.00	7.00	7.00	0.00	1.95	7.00	0.86	17.54	0.07	−0.56	0.13	0.08	1.08	162.00
MT	4.00	4.00	4.00	0.00	1.39	4.00	0.75	53.63	0.53	−0.81	0.13	0.07	1.08	32.00
MX	12.00	12.00	10.00	0.00	2.48	12.00	0.92	1.85	0.04	−0.91	0.12	0.06	1.06	19.00
SA	12.00	12.00	10.00	0.00	2.48	12.00	0.92	1147.21	0.34	0.81	0.03	0.29	1.28	3453.00
TU	8.00	8.00	8.00	0.00	2.08	8.00	0.88	8.68	0.05	−0.67	0.13	0.07	1.08	105.00
US	10.00	10.00	10.00	0.00	2.30	10.00	0.90	25.72	0.23	−0.81	0.12	0.06	1.07	38.00
Site														
CI1	4.00	4.00	4.00	0.00	1.39	4.00	0.75	5.19	0.03	−0.65	0.13	0.08	1.09	110.00
CI2	1.00	1.00	1.00	0.00	0.00	1.00	0.00	NA	NA	NA	0.13	NA	1.13	31.00
CY1	3.00	3.00	3.00	0.00	1.10	3.00	0.67	26.93	0.49	−0.86	0.13	0.07	1.09	38.00
EG3	3.00	3.00	3.00	0.00	1.10	3.00	0.67	0.05	0.00	−0.97	0.13	0.07	1.08	28.00
EG7	1.00	1.00	1.00	0.00	0.00	1.00	0.00	NA	NA	NA	0.13	NA	1.13	35.00
I1	1.00	1.00	1.00	0.00	0.00	1.00	0.00	NA	NA	NA	0.12	NA	1.12	16.00
I3	4.00	4.00	4.00	0.00	1.39	4.00	0.75	2.79	0.07	−0.95	0.13	0.07	1.08	12.00
MO1	3.00	3.00	3.00	0.00	1.10	3.00	0.67	21.80	0.64	−0.93	0.13	0.07	1.08	32.00
MO4	3.00	3.00	3.00	0.00	1.10	3.00	0.67	0.11	0.00	−0.69	0.13	0.07	1.09	89.00
MO5	1.00	1.00	1.00	0.00	0.00	1.00	0.00	NA	NA	NA	0.13	NA	1.13	30.00
MT1	1.00	1.00	1.00	0.00	0.00	1.00	0.00	NA	NA	NA	0.12	NA	1.12	18.00
MT2	3.00	3.00	3.00	0.00	1.10	3.00	0.67	−0.03	0.00	−0.96	0.13	0.06	1.08	14.00
MX	12.00	12.00	10.00	0.00	2.48	12.00	0.92	1.85	0.04	−0.91	0.12	0.06	1.06	19.00
SA1	2.00	2.00	2.00	0.00	0.69	2.00	0.50	NA	NA	0.49	0.03	0.08	1.06	340.00
SA2	4.00	4.00	4.00	0.00	1.39	4.00	0.75	15.92	0.18	0.12	0.01	0.01	1.01	146.00
SA3	3.00	3.00	3.00	0.00	1.10	3.00	0.67	0.32	0.00	0.28	0.06	0.10	1.10	530.00
SA4	3.00	3.00	3.00	0.00	1.10	3.00	0.67	3.08	0.28	−0.89	0.02	0.01	1.02	1758.00
TU2	4.00	4.00	4.00	0.00	1.39	4.00	0.75	5.85	0.05	−0.76	0.13	0.07	1.09	57.00
TU6	4.00	4.00	4.00	0.00	1.39	4.00	0.75	47.11	0.49	−0.80	0.13	0.07	1.08	34.00
US	10.00	10.00	10.00	0.00	2.30	10.00	0.90	25.72	0.23	−0.81	0.12	0.06	1.07	38.00

*Note:* Introduced, North Africa and the Mediterranean; Invaded, California and Mexico; Native, South Africa. NA values are recorded for sites where only one sample was represented.

Abbreviations: *AR*, allele richness; CI, Canary Islands; CY, Cyprus; EG, Egypt; *eMLG*, expected number of MLGs; $F_{\text{IS}}$, fixation index (inbreeding coefficient); *G*, Stoddard and Taylor's Index; *H*, Shannon–Weiner Diversity Index; $H_{s}$, expected heterozygosity; $H_{o}$, observed heterozygosity; I, Italy; *I*_*A*_, Index of Association; lambda, Simpson's Index; *MLG*, multilocus genotypes; MO, Morocco; MT, Malta; MX, Mexico; *N*, number of individuals; *PA*, private alleles; $\bar{r}d$, Standardised Index of Association; SA, South Africa; SE, rarefaction analysis standard error; TU, Tunisia; US, United States of America (California).

The country‐grouping scheme revealed similar diversity indices (*G*, *H* and lambda) and *MLG*s between the native and invaded ranges, but the standardised index of association in the native range was lower relative to four out of the seven introduced range countries (Table 3). The native South African and invaded Mexican populations both had similarly high measures of genetic diversity and *MLGs* (Table 3). Inbreeding (*F*_IS_), allele richness (*AR*), private alleles (*PA*) and observed and expected heterozygosity ($H_{o}$ and $H_{s}$) showed comparable output to that found under the broad grouping scheme.

### 3.4 ISSR Output

The HB13 primer yielded peaks that ranged in number between 0 and 27, with an average of 7 ± 8, and a total of 317 loci. The Jaccard error rate for HB13 was 0.72 ± 0.31. The HB15 primer yielded peaks that ranged in number between 0 and 35, with an average of 9.9 ± 11.1, and a total of 317 loci. The Jaccard error rate for HB15 was 0.69 ± 0.35.

Under the broad grouping scheme, the optimal *K*‐value was 2, while the country‐level grouping scheme showed *K* = 3 as being best suited to the data. The SplitsTree diagram showed a distinct grouping of native South African samples (Figure 5, clade D), although it contained one sample from Mexico. The introduced and invaded ranges were mixed across the remaining three clades (Figure 5, clades A–C).

The STRUCTURE output for the broader grouping scheme (*K* = 2) further supported this clustering pattern, where the native South African samples formed a separate genetic cluster (Figure 6). Selected samples from the Canary Islands, Crete, Cyprus, Morocco and Mexico matched the native South African cluster and, relative to the RADseq results, were likely misassigned. The ISSR data also did not detect South African site 4 (SA4) as being different from the other native sites (Figures 2 and 6).

The highest degree of within‐population genetic similarity (lowest genetic diversity) was observed within the invaded North American population (mean = 0.48 ± 0.35), and the lowest degree of similarity (highest genetic diversity) was observed within the native South African population (mean = 0.35 ± 0.37). The introduced range yielded a mean within‐population similarity value of 0.4 ± 0.31.

The invaded North American and introduced North African and Mediterranean populations shared the greatest degree of between‐population similarity (mean = 0.33 ± 0.27), while the native South African population was genetically distant from the introduced (mean similarity = 0.09 ± 0.08) and invaded (mean similarity = 0.11 ± 0.08) populations.

The AMOVA revealed that the highest proportion of genetic variation (98.51%) was found within samples (Table 4). A smaller proportion of the total variance (4.91%) was attributed to differences between broad groups, while the variation between countries was negligible (−3.42%), suggesting no meaningful genetic differentiation at the country level (Table 4). The Phi (Φ) statistic at the broad grouping level (Φ = 0.049) indicated low genetic structure between broad groups. Similarly, structuring between countries (Φ = −0.036) suggested no meaningful differentiation. The overall Phi statistic (Φ = 0.015) suggested that almost all variation was shared between individuals and that no meaningful population structure was present.

**TABLE 4:** Analysis of molecular variance (AMOVA) for *Mesembryanthemum nodiflorum* ISSR samples with two hierarchical grouping levels, showing the partitioning of genetic variation between and within groups.

Source of variation	df	σ	% variation
Between broad groups	2	150.73	4.91
Between countries within broad groups	8	−105.02	−3.42
Within samples	40	3026.15	98.51
Total	50	3071.85	100.00

*Note:* σ denotes the variation component.

## 4 Discussion

*Mesembryanthemum nodiflorum* has become invasive along the west coast of the United States of America and Mexico. The initiation of a management programme is underway to reduce the plant's growth and spread and aims to incorporate biological control as part of an integrated management approach (Olckers et al. 2021). The first step in establishing a biocontrol programme for this weed is to identify its source of origin so that native range surveys can be undertaken to find associated arthropods that display host‐specific feeding behaviour. Additionally, it is important to understand the weed's potential invasion pathways in order to fine‐tune management programmes (Hulme et al. 2008; Casso et al. 2019; Turbelin et al. 2022).

The present study used both RADseq and ISSR population genetic methods to assess the genetic structure and match the invasive *M. nodiflorum* populations in California and Mexico to populations in the native South African range and the introduced ranges of North Africa and the Mediterranean. Both population genetic methods showed that the invasive populations clustered with those from the introduced ranges of North Africa and the Mediterranean region, while the native South African range represented a separate, unique genetic cluster.

### 4.1 *Mesembryanthemum nodiflorum* Origin and Genetic Structure

The RADseq data provided support for a South African origin of *M. nodiflorum* based on its comparatively high number of private alleles, genetic isolation (high inbreeding) and allele richness. Considering the Wahlund effect in population genetics that results from different grouping approaches for the same data (De Meeûs 2018), we found expected genetic variation between sites within South Africa that were geographically separate. Site SA4 in Elands Bay, for example, showed evidence of being reproductively isolated, although the mechanisms behind this are not currently known. Compared with the positive inbreeding coefficients in the native range, the extent of negative values (i.e., outbreeding) found in the invaded and introduced ranges was surprising, although similar findings have been reported in the literature for invasive populations (Bossdorf et al. 2005; Genton et al. 2005; Storer et al. 2017; Kaňuch et al. 2021). It is likely that the human‐aided dispersal of *M. nodiflorum* to the Mediterranean accelerated genetic admixture in the region.

Although *M. nodiflorum* is listed as being native to the westernmost area of the Mediterranean region in some sources (Biota 2025), it is more likely a historical introduction through both natural patterns and human dispersal. It is also possible that the eastern Mediterranean region was colonised independently at a later stage by means of trade via the Suez Canal. Two root‐feeding weevils in the genus *Temnorhinus* (Coleoptera: Curculionidae), considered native to the Canary Islands but also found in the Mediterranean Basin (Oromí 2018), were recently found causing damage on *M. nodiflorum* in the Canary Islands (pers.comm. M. Cristofaro). One of these weevils, *Temnorhinus mixtus* (F.), has been shipped to the USDA‐ARS in California, USA for study in quarantine. It is possible that this weevil has adopted *M. nodiflorum* as a host, and its host range encompasses other plants in the same arid, sandy habitats.

The Mediterranean Basin is a major historic source of secondary plant invasions, where regular exchanges have been recorded between the region and other Mediterranean‐like climates that include South Africa, Chile, California and southern Australia (Fox 1990; Brunel et al. 2010). Fox (1990) mentions that before the opening of the Suez Canal in 1869, trading ships bound for Europe and England from Australia passed the Cape of Good Hope in South Africa. It was encouraged at the time to collect exotic plant material, which could have been one of many likely paths for *M. nodiflorum* 's northward dispersal. Multiple independent prior introductions from South Africa to the Mediterranean are also likely, contributing to the lower degree of inbreeding observed in the introduced populations that would have arrived later in North Africa. The endemic southern African crystalline iceplant, *Cryophytum crystallinum* L. (Aizoaceae), which similarly invades the Californian coast, was also reportedly first introduced to the eastern Mediterranean before being transported to England, where it arrived by 1725 (Kloot 1983). *Cryophytum crystallinum* arrived in southern Australia by 1851 and became widespread by 1879 (Kloot 1983). As an estimate, *M. nodiflorum* is likely to have followed a similar invasion trajectory.

The present results also revealed some evidence of varying reproductive strategies, where, on average, asexual reproduction appears to occur more frequently in the native range and in some of the introduced range populations, relative to the invaded range. A flexible reproductive strategy is known to occur in many invasive plant populations, where switches between sexual and asexual reproduction at different stages of the invasion can be a key contributor to successful establishment and spread (Dong et al. 2006; Barrett et al. 2008; Burns et al. 2013). Jesse et al. (2010), for example, found that some patches of invasive *Rosa multiflora* Thunb. populations in the eastern USA established by seed, spread clonally, and after reaching a larger population size, switched back to sexual reproduction to augment genetic diversity. Invasive *M. nodiflorum* populations might be following a similar process, switching between amphimictic and apomictic seed production. *Mesembryanthemum nodiflorum* reproduces by seed only, which can remain dormant for decades (Gutterman and Gendler 2005).

### 4.2 Comparative Output of RADseq and ISSR Techniques

The ISSR analysis broadly agreed with the RADseq output when examined at the optimal *K*‐values, but was not useful for investigating further population‐level structuring (see also Lemopoulos et al. (2019) for a comparison of RADseq and microsatellite data). While the outcomes of both the ISSR and RADseq analyses revealed similar patterns, the results obtained through the RADseq analysis provided more insight into the potential invasion pathways of *M. nodiflorum* , greater confidence in assigning individuals to putative populations and revealed population structure within the native range at a higher resolution.

Although RADseq outperforms fragment analysis in terms of data resolution, particularly with smaller sample sizes, the choice of genetic marker depends on the specific investigative questions and available resources. The predominant benefit of a RADseq approach is that it is reproducible across laboratories and produces information‐rich SNP data. Comparatively, ISSRs are typically not reproducible across laboratories due to both the subjectivity of scoring parameters and differences in reagents and equipment (Bonin et al. 2004), and produce only binary data output. The need for ISSR sample replication can become expensive for large sample sizes, and high error rates could preclude a large portion of samples from downstream analyses. These factors should be taken into account before commencing with a research project.

### 4.3 Implications for Biological Control

Since our results provide further confirmation that South Africa is the native origin of *M. nodiflorum* , surveys for potential biological control agents should focus on the plant's native range in southern Africa. Native southern African plants likely spread to the Mediterranean, where these introduced populations remained for some time before being transported to North America. Bridgehead populations are widely reported in the literature, serving as sources of secondary invasions (De Kort et al. 2016; Barker et al. 2017; Bertelsmeier and Keller 2018; Lesieur et al. 2019; Blumenfeld et al. 2021; Vallejo‐Marín et al. 2021). The diversity of specialist natural enemies will be greatest in the indigenous distribution, and so the southern African range is where promising candidate agents are most likely to be sourced. Fewer specialist natural enemies are expected in the Mediterranean, but if present, they are likely to be adapted to the plant genotypes that occur in the invaded distribution of California and Mexico and should therefore also be considered as potential biocontrol agents.

Considering the high level of genetic diversity found in the indigenous distribution, more sampling in South Africa is likely to uncover additional unique *M. nodiflorum* populations. It is therefore possible that new genotypes would match the alien populations better.

## 5 Conclusion

This study has confirmed that the native distribution of *M. nodiflorum* is in South Africa, and that is where the highest diversity of potential biological control agents is likely to be found. However, the close relationship between the Mediterranean populations and the invasive populations in North America also suggests that any natural enemies present in that area could also be considered. Specialist natural enemies found in the Mediterranean will most likely have been introduced from southern Africa. The discovery of potential biocontrol agents for *M. nodiflorum* offers exciting prospects for the management of this weed and the restoration of invaded habitats.

## Author Contributions

**Clarke J. M. van Steenderen:** data curation (lead), formal analysis (lead), investigation (equal), methodology (lead), software (lead), validation (lead), visualization (lead), writing – original draft (lead), writing – review and editing (lead). **Emma Sandenbergh:** data curation (equal), formal analysis (supporting), investigation (equal), writing – review and editing (equal). **Dean Brookes:** methodology (equal), supervision (equal), writing – review and editing (equal). **Patrick J. Moran:** conceptualization (lead), data curation (supporting), funding acquisition (lead), resources (lead), writing – review and editing (equal). **Massimo Cristofaro:** data curation (equal), writing – review and editing (equal). **William F. Hoyer III:** conceptualization (equal), funding acquisition (equal), writing – review and editing (equal). **Iain D. Paterson:** conceptualization (lead), funding acquisition (lead), project administration (lead), resources (lead), supervision (lead), writing – review and editing (equal).

## Conflicts of Interest

The authors declare no conflicts of interest.

## Supporting information


**Data S1:** ece372262‐sup‐0001‐Supinfo01.xlsx.

## Data Availability

All data and code required to reproduce the analyses are available at both the public GitHub repository https://github.com/clarkevansteenderen/RADseq_pipeline and the permanent Zenodo doi: 10.5281/zenodo.17044994. RADseq data files are available on the SRA database (https://www.ncbi.nlm.nih.gov/sra) under project ID PRJNA1284890 and are also housed on the CBC's data repository.

## Appendix A Methods Tables

**TABLE A1:** Restriction digestion PCR constituents.

Reagent (TruSeq)	1× (uL)
NEB 10× CutSmart buffer	1.5
dH_2_O	4.5
MspI [20.000 U/mL] (Read 1 enzyme)	0.5
EcoRI‐HF [20.000 U/mL] (Read 2 enzyme)	0.5
ClaI [10.000 U/mL] (Read 1 adapter dimer‐cutting enzyme)	1.0
**Total**	**8.0**
iTru ClaI adapter [5 μM] (Read 1 adapter) in eight unique tags	1.0
iTru EcoRI adapter [5 μM] (Read 2 adapter) in 12 unique tags	1.0
Genomic DNA (normalised)	5.0
**Final volume**	**15.0**

**TABLE A2:** Ligation PCR constituents.

Reagent	1× (uL)
${\mathrm{dH}}_{2}O$	2.75
rATP (10 mM)	1.5
10× ligase buffer	0.5
DNA T4 ligase [400 units/μL]	0.25
**Final volume**	**5.0**

**TABLE A3:** Library preparation PCR constituents.

Reagent	1× (uL)
Nuclease‐free water	8.5
5× Phusion HF buffer	10
10 mM dNTPs	1
5 μM forward primer (iTru5_01_A)	5
5 μM reverse primer (iTru7_101_01)	5
**Pooled DNA sample**	**20**
DMSO (optional)	0
Phusion DNA polymerase	0.5
**Final volume**	**50**

**TABLE A4:** PCR protocol for the ISSR HB13 and HB15 primers.

Cycles	Step	Temperature (°C)	Time
1	Initial denaturation	95	7 min
38	Denaturation	95	45 s
	Annealing	44 (HB13); 48 (HB15)	45 s
	Extension	72	2 min
1	Final extension	72	7 min
1	Hold	12	∞
